# Supplementary material for: Impaired regulatory T-cell–mediated immune tolerance promotes neurodegeneration in glaucoma
Source: medRxiv. 2026 Sep 20:2026.09.17.26363354. Preprint. [Version 1] doi: 10.64898/2026.09.17.26363354 (PMC13596698; doi:10.64898/2026.09.17.26363354)
Supplement: Supplementary file 1 [file media-1.pdf]

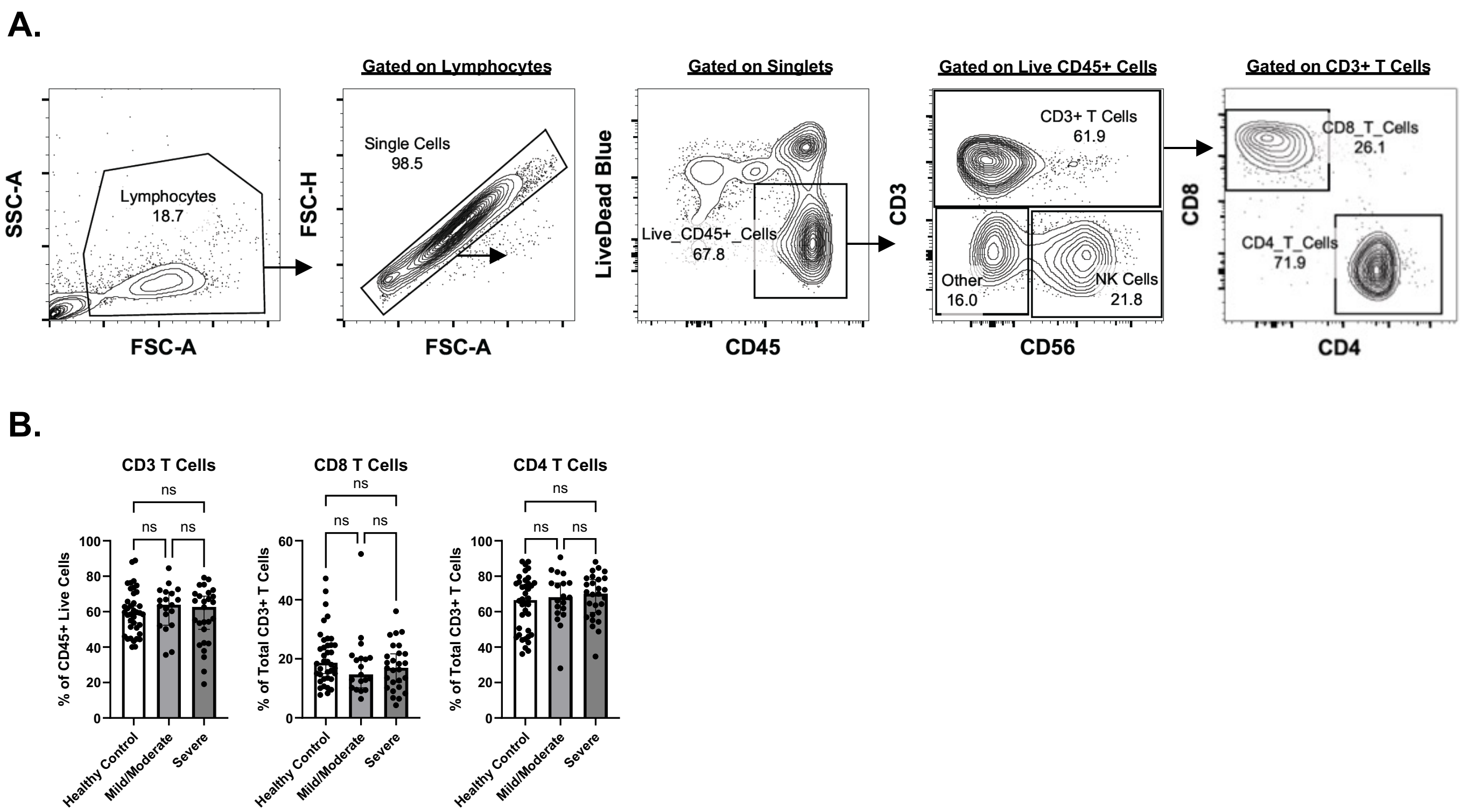

Supplementary Figure 1: Flow cytometry gating strategy and peripheral T cell frequencies across disease severity groups. (A) Representative flow cytometry gating strategy for peripheral blood mononuclear cells (PBMCs). Lymphocytes were first identified based on forward scatter (FSC-A) and side scatter (SSC-A). Doublets were excluded by gating on single cells (FSC-A vs. FSC-H). Live $CD45^+$ leukocytes were selected using viability dye exclusion, followed by identification of $CD3^+$ T cells. $CD3^+$ cells were further subdivided into $CD4^+$ and $CD8^+$ T cell populations. Representative percentages for each gate are shown within the plots. (B) Quantification of $CD3^+$ T cells (% of $CD45^+$ live cells), $CD8^+$ T cells (% of total $CD3^+$ T cells), and $CD4^+$ T cells (% of total $CD3^+$ T cells) in healthy controls, mild/moderate disease, and severe disease groups. Data are presented as mean ± SEM with individual data points shown. Statistical analysis via One-way ANOVA. No statistically significant differences were observed among groups (ns, not significant).
