# Supplementary material for: Impaired regulatory T-cell–mediated immune tolerance promotes neurodegeneration in glaucoma
Source: medRxiv. 2026 Sep 20:2026.09.17.26363354. Preprint. [Version 1] doi: 10.64898/2026.09.17.26363354 (PMC13596698; doi:10.64898/2026.09.17.26363354)
Supplement: Supplementary file 3 [file media-5.pdf]

A.

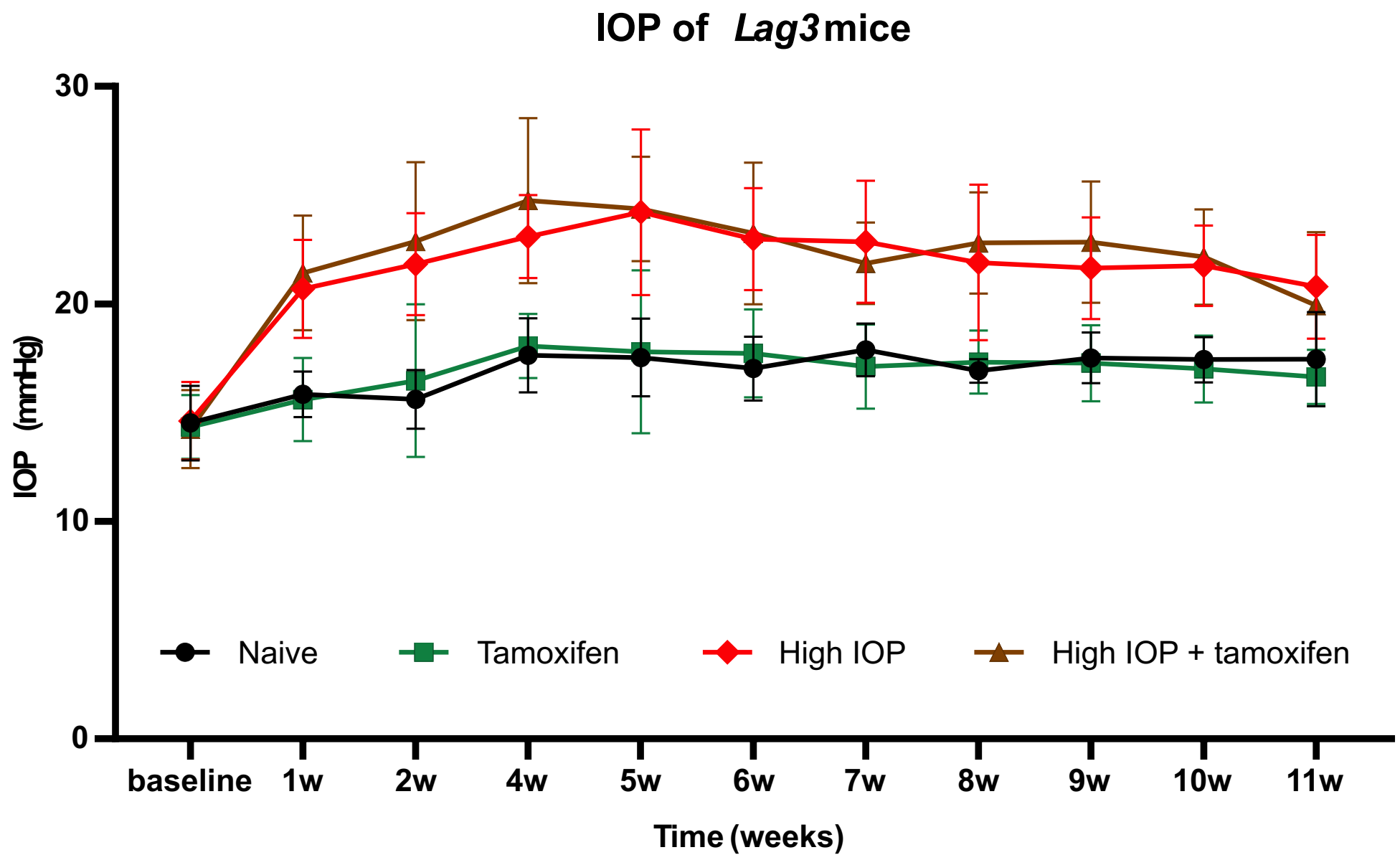

Supplementary Figure 5: Intraocular Pressure of Lag3$^{fl/fl}$.Foxp3Cre$^{ERT2\text{-}GFP}$ Mice Over Time. (A) Intraocular pressure (IOP) was measured longitudinally at baseline and weekly for 11 weeks in naïve, tamoxifen-only, High IOP, and Tamoxifen + High IOP groups. Naïve and Tamoxifen-only mice maintained stable IOP levels throughout the study period. In contrast, High IOP-treated mice exhibited a rapid increase in IOP beginning at 1 week, peaking around 4–5 weeks, and remaining elevated compared to controls. Data are presented as mean ± SEM (mmHg).
